# Monitoring Endocrine Complications of Immunotherapy: A Screening Tool

**DOI:** 10.7759/cureus.26859

**Published:** 2022-07-14

**Authors:** Priyanka Majety, Anna Groysman, Virginia Seery, Meghan Shea, Runhua Hou

**Affiliations:** 1 Endocrinology, Diabetes, and Metabolism, Beth Israel Deaconess Medical Center, Harvard Medical School, Boston, USA; 2 Medical Oncology, Beth Israel Deaconess Medical Center, Harvard Medical School, Boston, USA

**Keywords:** immune-related adverse events, immunotherapy, screening tool, endocrinopathies, immune checkpoint inhibitors

## Abstract

Introduction

The advent of immunotherapy has revolutionized cancer therapy in recent years. Immunotherapy using monoclonal antibodies against checkpoint molecules, including programmed death (PD)-1, PD ligand (PD-L)1, and cytotoxic T-lymphocyte antigen 4 (CTLA)-4, has become a cornerstone in cancer therapy. However, due to the physiologic role of checkpoint molecules in preventing autoimmunity, immune-related adverse events (irAEs) have emerged as frequent complications. As the use of immunotherapy increases, a better understanding of irAEs and screening tools for timely diagnosis and management are needed.

Materials and methods

We surveyed oncology providers at our institution with 10 questions assessing their knowledge, and comfort levels in diagnosing and managing endocrine irAEs. We created an endocrine clinic referral order specifically for oncology-related endocrinopathies and created a screening tool for diagnosing these endocrinopathies. We met with our oncology providers in three different hour-long sessions. A post-intervention survey was sent out six months after our initial meeting to assess changes in the participants’ knowledge and comfort levels. We also reviewed the electronic medical records system for the number of new referrals to endocrinology clinic.

Results

A total of 27 (N) participants responded to the initial survey and 14 (n) responded to the subsequent survey six months later. Based on the initial survey, only a minority (26%) of respondents were comfortable diagnosing and managing (15%) immunotherapy-related adrenal dysfunction whereas more respondents were comfortable diagnosing (55%) and managing (56%) thyroid dysfunction. The majority (67%) of the respondents knew which immunotherapies commonly are implicated in hypophysitis but only 42% of them were aware of the next steps of its management. We noted a significant increase in self-reported comfort levels in diagnosing (p < 0.05) and managing (p < 0.05) adrenal disorders post-intervention. There was also a trend of improvement in participants’ comfort levels regarding diagnosing and managing thyroid dysfunction, management of hypophysitis, and immunotherapies implicated in thyroid dysfunction but the changes did not reach statistical significance. There was no significant change in their knowledge regarding immunotherapies implicated in hypophysitis and natural history of thyroid dysfunction in this setting. In the six months following our intervention, 30% (n=21) of the patients referred to the endocrine clinic were for immune-related endocrinopathies compared to 19% (n=7) of patients over a similar duration before the intervention. Data on the time between referral and endocrinology appointment was available for 16 out of the 21 patients and the mean (±SD) time to endocrine clinic appointment was 2.66 (±1.95) weeks. Nine (43%) of the 21 referred patients were seen in endocrinology clinic within two weeks.

Conclusions

Although immune-related endocrinopathies are rarely fatal, they have a significant impact on patients’ quality of life. Endocrinopathies are typically manageable with prompt recognition and treatment. But the subtle and non-specific manifestations make the diagnostic process a challenge. Standardized and practical screening tools can help in diagnosing these adverse events promptly, seeking specialized care if needed and may also aid in reducing healthcare-related costs.

## Introduction

The advent of immunotherapy has revolutionized cancer therapy. Immune checkpoint inhibitors (ICIs) are a group of monoclonal antibodies that commonly target the receptors cytotoxic T-lymphocyte antigen 4 (CTLA-4), and programmed cell death protein 1 (PD-1) or its associated ligand (PD-L-1). By attenuating these pathways, the ICIs deprive cancer cells of a key strategy of evasion from immunosurveillance. Antibodies that bind to CTLA-4, PD-1, and PD-L1 lead to T-cell activation and destruction of both tumor and normal host cells. At the same time, ICI unleashes T cells to fight cancer and they also trigger autoimmune manifestations in different organs, including endocrine organs, generally referred to as immune-related adverse events (irAEs) [1].

Use of ICIs, as both single and combination agent therapies, in cancer treatment has increased vastly over the past decade and is now an established cornerstone for cancer therapy. Currently, there are three FDA-approved PD-1 inhibitors, three PD-L1 inhibitors, one CTLA-4 inhibitor, and one lymphocyte activation gene-3 (LAG-3) inhibitor. Their use is estimated to increase as newer checkpoint inhibitors are being approved for cancer management [2]. The estimated percentage of patients in the United States with cancer who are eligible for checkpoint inhibitor drugs increased from 1.54% in 2011 to 43.63% in 2018 and the percentage of patients estimated to respond to checkpoint inhibitor drugs increased from 0.14% to 12.46% [3]. Depending on the immunotherapy agent used, immune-related endocrinopathies affect about 40% of the treated patients [4]. 

Currently, there are guidelines from professional societies like The American Society of Clinical Oncology (ASCO) [5], Society of Immunotherapy for Cancer (SITC) [6], National Comprehensive Cancer Network (NCCN) [7], European Society of Medical Oncology (ESMO) [8] and Society for Endocrinology Clinical Committee [9] for management of all immune-related adverse events, which provide advice on laboratory monitoring and treatment of these adverse events. Given the increasing use of ICIs, the life-threatening nature of endocrine dysfunction, and the rising need for inpatient admissions for their management, we aimed to increase the awareness of these complications, develop an institutional practical screening tool, increase the comfort levels of oncologists diagnosing them, and streamline the referral process to endocrinology for prompt management.

Portions of the abstract discussed in this article were previously presented as an e-poster at the 2022 American Association of Clinical Endocrinology annual meeting on May 13, 2022.

## Materials and methods

After obtaining institutional IRB approval (protocol #2021P000293), we surveyed oncology providers (attendings, nurse practitioners, fellows) at our institution with a 10-question survey assessing their knowledge and comfort levels in diagnosing and managing endocrine irAEs (Appendices). Based on their responses, we curated a presentation focused on identifying the common presenting symptoms of endocrinopathies, diagnosis, and their immediate management. We created an endocrine clinic referral order specifically for oncology-related endocrinopathies in our electronic medical records system. In addition, with the help from both divisions of endocrinology and medical oncology, we created our institutional screening tool for diagnosing immune-related endocrinopathies, based on the currently available guidelines (Tables 1, 2). This also included indications for referral to endocrinology (Tables 1, 2) and how to interpret common endocrine labs (Table 3).

**Table 1:** Monitoring thyroid dysfunction and hyperglycemia with PD1 and PDL1 blockade TSH: thyroid-stimulating hormone; FT4: free thyroxine; TPOAb: thyroid peroxidase antibody; ACTH: adrenocorticotropin hormone; PD1: programmed death 1; PDL1: programmed death ligand 1

Time	Testing Interval	Tests	Indications for Referral to Endocrinology and Notes
Baseline	Before starting	TSH, FT4, glucose, TPOAb	( ) TSH < 0.01, high FT4, pattern persists after repeat testing (for management of hyperthyroidism)
0-6 months	Before each cycle	TSH, FT4, glucose	( ) Low/ normal TSH, low FT4: obtain 8 AM cortisol and ACTH (for diagnosis of central hypothyroidism vs. euthyroid sick syndrome and rule out secondary adrenal insufficiency)
6-12 months	Every 3 months	TSH, FT4, glucose	( ) Glucose > 250 mg/dL - acute onset of polyuria, polydipsia, weight loss, anion gap > 10, HCO_3_≤18, arterial PH≤ 7.30, positive ketones in urine and serum (for diagnosis and management of diabetic ketoacidosis)
>12 months	Every 6 months	TSH, FT4, glucose	( ) TPOAb (optional at baseline only if there is family history of thyroid disease)

**Table 2:** Monitoring endocrinopathies with combination therapy or CTLA-4 inhibitor monotherapy TSH: thyroid-stimulating hormone; FT4: free thyroxine; TPOAb: thyroid peroxidase antibody; ACTH: adrenocorticotropin hormone; CTLA-4: cytotoxic T-lymphocyte antigen 4

Time	Testing Interval	Tests	Indications for Referral to Endocrinology and Notes
Baseline	Before starting	TSH, FT4, glucose, cortisol, TPOab	( ) 8 AM cortisol < 10 or random cortisol < 3 (for diagnosis of primary or secondary adrenal insufficiency); consider MRI of pituitary if severe headache, nausea, vomiting, visual changes.
0-6 months	Before each cycle	TSH, FT4, glucose, cortisol	( ) TSH < 0.01, high fT4, pattern persists after repeat testing (for management of hyperthyroidism)
6-12 months	Every 3 months	TSH, FT4, glucose, cortisol	( ) Low/normal TSH, low FT4: obtain 8 AM cortisol and ACTH (for diagnosis of central hypothyroidism vs. euthyroid sick syndrome and rule out secondary adrenal insufficiency)
>12 months	Every 6 months	TSH, FT4, glucose	( ) Glucose > 250 mg/dL - acute onset of polyuria, polydipsia, weight loss, anion gap > 10, HCO_3_≤18, arterial PH≤ 7.30, positive ketones in urine and serum (for diagnosis and management of diabetic ketoacidosis)
	Every 12 months	Cortisol	( ) TPOAb (optional; only at baseline if there is family history of thyroid disease ); ( ) if random cortisol is <3 or symptomatic, send 8 AM cortisol and ACTH for confirmation; ( ) cortisol interpretation is dependent on any recent use of systemic steroids and skip cortisol test if currently on steroid.

**Table 3:** Interpretation of common endocrine laboratory tests TSH: thyroid-stimulating hormone; FT4: free thyroxine; TPOAb: thyroid peroxidase antibody; ACTH: adrenocorticotropin hormone; TgAb: thyroglobulin antibody; TRAb: TSH receptor autoantibody

Endocrine Tests	Interpretation
Low ACTH, low cortisol	Secondary adrenal insufficiency
	( ) Consider pituitary MRI
	( ) Consider withholding therapy if symptoms of severe hyponatremia/headache/visual disturbance/cranial nerve palsy are present
High ACTH, low cortisol	Primary adrenal insufficiency
High TSH, low FT4	Primary hypothyroidism
	( ) Evaluate TPOAb, if negative, obtain TgAb
Low/normal TSH, low FT4	Secondary hypothyroidism
	( ) Evaluate for secondary adrenal insufficiency and obtain pituitary MRI prior to initiation of levothyroxine
Low TSH, high FT4	Thyrotoxicosis
	( ) Monitor TFTs every 2-3 weeks for development of hypothyroidism or persistent thyrotoxicosis
	( ) Obtain radioactive iodine (RAI) uptake and scan or obtain TRAb, TgAb, and TPOAb, if RAI uptake and scan not possible

We met with oncology physicians, nurse practitioners, and fellows, respectively, in three different virtual hour-long sessions to introduce the screening tool, referral order set and discussed the diagnosis and management of endocrinopathies with the help of case-based scenarios. The protocol was further disseminated on the oncology division’s website and via e-mail.

A post-test survey was sent out six months after our initial meeting to assess changes in the participants’ knowledge and comfort levels. We also reviewed the electronic medical records system for the number of new referrals to endocrinology clinic for immune-related endocrinopathies. The association between categorical variables was performed using chi-square test. All statistical analyses were performed using Microsoft Excel and chi-square calculator [10]. A p-value < 0.05 was considered statistically significant.

## Results

A total of 27 (N) participants responded to the initial survey. Of them, 10 (37%) were attending physicians, eight (30%) were nurse practitioners, and nine (33%) were fellows. Only a minority (26%) of the respondents stated that they are comfortable diagnosing (26%) and managing (15%) immunotherapy-related adrenal dysfunction whereas more respondents were comfortable diagnosing (55%) and managing (56%) thyroid dysfunction. The majority (67%) of the respondents knew which immunotherapies commonly are implicated in hypophysitis but only 42% of them were aware of the next steps of its management. Only a small portion of respondents was aware of the common immunotherapies that lead to thyroid dysfunction (26%) and its natural history (35%).

We surveyed the participants six months after our presentation and introduction of the screening tool. A total of 14 (n) responded to the post-test. Of them, six (43%) were attending physicians, three (21%) were nurse practitioners, and five (36%) were fellows. We noted a significant increase in self-reported comfort levels in diagnosing (p=0.005) and managing (p=0.016) adrenal disorders (Table 4). 

**Table 4:** Comparing comfort levels and knowledge of participants before and after intervention *Significant change, p-value <0.05.

Comfort Levels and Knowledge of Participants	Pre-test (N=27) % (Number)	Post-test (n=14) % (Number)	p-Value
Comfortable diagnosing adrenal complications*	26% (7)	71 % (10)	0.005*
Comfortable managing adrenal complications*	15% (4)	50% (7)	0.016*
Comfortable diagnosing thyroid complications	55% (15)	79% (11)	0.147
Comfortable managing thyroid complications	55% (15)	79% (11)	0.147
Immunotherapies implicated in hypophysitis	67% (18)	79% (11)	0.427
Management of hypophysitis	41% (11)	64% (9)	0.153
Immunotherapies implicated in thyroid dysfunction	26% (7)	50% (7)	0.123
Natural history of thyroid dysfunction	33% (9)	29% (4)	0.756

There was also a trend of improvement in participants’ comfort levels regarding diagnosing (55-79%) and managing (55-79%) thyroid dysfunction in the setting of immunotherapy use, management of hypophysitis (41-64%), and immunotherapies implicated in thyroid dysfunction (26-50%), although statistical difference was not achieved. There was no significant change in their knowledge regarding natural history of thyroid dysfunction in this setting (p=0.756) and immunotherapies implicated in hypophysitis (p=0.427).

In the six months following our intervention, 21 (28%) of 74 endocrinology consults were for endocrinopathies related to immune checkpoint inhibitors, compared to seven (19%) of 37 consults over a similar duration before the intervention (p=0.279). Data on the time between referral and endocrinology appointment were available for 16 out of the 21 patients and the mean (±SD) time to endocrine clinic appointment was 2.66 (±1.95) weeks. Nine (43%) of the 21 referred patients were seen in endocrinology clinic within two weeks.

## Discussion

Immune checkpoint inhibitors have received broad and significant interest because of their ability to generate durable responses in intractable malignant tumors and for improvements in overall survival [11,12]. The number of clinical trials using PD-1 and PD-L1 inhibitors has increased by nearly 600% between just 2015 and 2017 [13] and the market is expected to grow similarly [10]. Currently, the most used ICIs are CTLA-4 inhibitors (ipilimumab), PD-1 inhibitors (pembrolizumab and nivolumab), and PD-L1 inhibitors (atezolizumab, avelumab, and durvalumab).

Initiation of checkpoint inhibitor therapy is frequently associated with irAEs. Multiple organs can be affected by immune-mediated reactions including colitis, hepatitis, dermatitis, and pneumonitis among others, in addition to endocrinopathies. Immune-related endocrinopathies affect about 40% of the treated patients [14]. Most common endocrinopathies include autoimmune thyroid dysfunction (usually hypothyroidism which may be preceded by thyroiditis-induced thyrotoxicosis), hypophysitis, primary adrenal insufficiency, insulin deficient diabetes mellitus [15,16], and rarely hypoparathyroidism [17,18]. Incidence of endocrinopathies varies based on the checkpoint inhibitor used [16]. While typically the symptoms present within five to six months of initiation of immunotherapy, their onset is highly variable and can even occur months after discontinuation of therapy [19]. Thyroid dysfunction is more common in patients treated with combination therapy (CTLA-4 and PD-1 inhibitors) followed by PD-1/PD-L1 inhibitors and CTLA-4 inhibitors [16]. Whereas hypophysitis and adrenal insufficiency occur more frequently in patients treated with combination therapy followed by CTLA-4 inhibitors and PD-1/PD-L1 inhibitors. Insulin deficient diabetes mellitus was more common with combination therapy followed by PD-1/PD-L1 inhibitor use [16].

These endocrinopathies can occur in an unpredictable fashion and can be a source of significant and persistent morbidity and rarely can be fatal [20]. Unlike other irAEs, endocrine irAEs tend to be irreversible necessitating lifelong treatment. Supportive treatment with hormone replacement is the key. Due to the rarity and vagueness of the presenting symptoms, there can be a delay in diagnosing immune-related endocrinopathies. If untreated, they lead to increased hospitalizations for management of these adverse events [21]. It has been shown that the patients requiring inpatient admission for management of irAEs have significantly higher percentage of readmission, all-cause in-hospital mortality, length of stay, and lower retention of ICI therapy [22]. This contributes significantly to healthcare-related costs. The average total cost of medical care after a diagnosis of hypophysitis is estimated to be $16,465 whereas the average cost associated with diabetes, thyroid dysfunction, and adrenal insufficiency is $9,453, $9,399, and $3,974, respectively. The overall mean (±SD) cost was $29,477 (± $48,087) for patients with an inpatient baseline irAE-related visit and $5,718 (± $13,720) for patients with a baseline irAE-related outpatient visit [22].

In our study, we noted an improvement in the comfort levels of oncology providers in diagnosing and managing these complications. In addition, there was a substantial increase in the number of referrals to our endocrinology clinic for specialized care, and timely outpatient care was provided. While our screening tool is easy to follow and can be easily adapted to the needs of any healthcare facility, our study has several limitations. This study was done at a single institution and the number of participants in our study was low. Due to the pandemic, there was a high turnover of employees at our hospital which has contributed to low post-test response rate. The other likely reasons for non-participation were lack of time and the survey nature of the study. The follow-up period has only been six months. We aim to periodically meet with the division of oncology and update our protocol based on the latest evidence given this is a quality improvement project and continued modification of our protocol is warranted based on clinical need. More open access screening protocols should be made available for clinical use. We were unable to assess the impact of our screening tool on inpatient admissions for immune-related endocrinopathies. As a result, we were unable to assess its cost-effectiveness at this time. Finally, due to the anonymous nature of the questionnaires, paired analysis of the responses was not possible. We plan to introduce the screening protocol to other hospitals within our network in the near future so this study could be replicated in a larger cohort. Future studies should look at the performance of these protocols in larger patient populations and their effects on health care-related costs, in addition to their cost-effectiveness.

## Conclusions

Although immune-related endocrinopathies are rarely fatal, they have a significant impact on patients’ quality of life. They are different from other irAEs as they result in permanent, irreversible endocrine dysfunction. Immune-related endocrinopathies do not typically require discontinuation of immunotherapy or high-dose steroids for immunosuppression. Supportive management with hormone replacement is the key to achieving optimal patient outcomes and these patients generally require lifelong hormone supplementation.

Endocrinopathies are typically manageable with prompt recognition and treatment. But the subtle and non-specific manifestations, especially since these presenting symptoms can overlap with cancer-related or cancer therapy-related complications (e.g., nausea which can be a presenting symptom of adrenal insufficiency can also occur as a side effect of chemotherapy or underlying cancer, both hypophysitis and opiates can cause loss of libido, depression can be a manifestation of the cancer diagnosis itself or hypothyroidism), make the diagnostic process a challenge. Due to these reasons, a high clinical index of suspicion is required by the treating oncologists or primary care physicians. Therefore, regular symptom monitoring and screening for endocrinopathies are important. Patients who have positive screening tests may require timely referral to endocrinology for management. Standardized and practical screening tools can help diagnose these adverse events promptly, seek specialized care if needed, and may also aid in reducing healthcare-related costs.

## Appendices

Questionnaire used to assess participants' comfort levels and knowledge with immune-related endocrinopathies

Monitoring Immune-Related Endocrinopathies - Questionnaire

1. Which of the following immune-checkpoint inhibitors is most likely to cause hypophysitis?

a) Ipilimumab (CTLA-4 inhibitor)

b) Nivolumab - Ipilimumab (combination of CTLA-4 and PD-1 inhibitors) (correct answer)

c) Pembrolizumab (PD-1 inhibitor)

d) Atezolizumab (PD-L1 inhibitor)

e) I don’t know

2. Which of the following immune-checkpoint inhibitors is most likely to cause thyroid-related dysfunction?

a) Ipilimumab (CTLA-4 inhibitor)

b) Atezolizumab (PD-L1 inhibitor)

c) Pembrolizumab (PD-1 inhibitor) (correct answer)

d) I am not sure/ I don’t know

3. In patient who was treated with immune-checkpoint inhibitor therapy, TFTs show a low free T4 level and low TSH level along with symptoms of hypothyroidism but no headache, visual changes, nausea, what is the next best step:

a) Order STAT pituitary MRI

b) Start Levothyroxine at 1.6 mcg/Kg daily on an empty stomach

c) Check 8AM cortisol & ACTH (adrenocorticotropin hormone) (correct answer)

d) Recheck thyroid function tests in 3 weeks

e) I am not sure/ I don’t know

4. Which of the following is the more common thyroid-related dysfunction in patients treated with immune-checkpoint inhibitors?

a) Primary hypothyroidism

b) Secondary hypothyroidism

c) Thyrotoxicosis followed by primary hypothyroidism (correct answer)

d) Graves’ disease

e) I am not sure/ I don’t know

5. Which of the following is FALSE regarding testing cortisol in a patient?

a) Cortisol level is affected by exogenous steroids

b) Cortisol level is not affected by opioid pain medications (correct)

c) The timing of cortisol testing is important, as it has a diurnal rhythm

d) I am not sure/ I don’t know

6. Which of the following immune-checkpoint inhibitors is most likely to pancreatic insufficiency, or diabetic ketoacidosis?

a) Pembrolizumab (correct answer)

b) Ipilimumab

c) Tremelimumab

d) I am not sure/ I don’t know

7. How comfortable do you feel about diagnosing immune-checkpoint-related thyroid dysfunction?

a) Very comfortable

b) Comfortable

c) Neutral

d) Uncomfortable

e) Very uncomfortable

8. How comfortable do you feel about managing immune-checkpoint-related thyroid dysfunction?

a) Very comfortable

b) Comfortable

c) Neutral

d) Uncomfortable

e) Very uncomfortable

9. How comfortable do you feel about diagnosing immune-checkpoint-related adrenal dysfunction?

a) Very comfortable

b) Comfortable

c) Neutral

d) Uncomfortable

e) Very uncomfortable

10. How comfortable do you feel about managing immune-checkpoint-related adrenal dysfunction?

a) Very comfortable

b) Comfortable

c) Neutral

d) Uncomfortable

e) Very uncomfortable
